# High-resolution peripheral quantitative computed tomography imaging and bone biomarker characteristics of patients with ischemic stroke or transient ischemic attack—baseline analysis of the Post-stroke Osteopathy study

**DOI:** 10.1007/s11657-026-01767-w

**Published:** 2026-08-29

**Authors:** Benjamin Dejakum, Johannes Bereiter-Payr, Silvia Felicetti, Kurt Moelgg, Anel Karisik, Gerald Degenhart, Thomas Toell, Christian Boehme, Lukas Mayer-Suess, Raimund Pechlaner, Anna-Maria Liphardt, Gabriela Berg, Georg Schett, Stefan Kiechl, Michael Knoflach

**Affiliations:** 1 Department of Neurology, Medical University of Innsbruck, Anichstrasse 35, 6020 Innsbruck, Austria; 2 VASCage-Centre On Clinical Stroke Research, Innsbruck, Austria; 3 Core Facility MicroCT, Medical University of Innsbruck, Innsbruck, Austria; 4 Department of Internal Medicine 3-Rheumatology and Immunology, Friedrich-Alexander-Universität Erlangen-Nürnberg, Universitätsklinikum Erlangen, Erlangen, Germany; 5 Deutsches Zentrum Immuntherapie, Friedrich-Alexander-Universität Erlangen-Nürnberg, Universitätsklinikum Erlangen, Erlangen, Germany; 6 Biomedica Medizinprodukte GmbH, Vienna, Austria

**Keywords:** Stroke, Fractures, HR-pQCT, Biomarkers

## Abstract

***Summary*:**

Stroke or transient ischemic attack (TIA) leads to unfavorable structural changes in the bones. The underlying pathophysiological process is unclear. We provide a detailed characterization of the structural and biochemical properties of bone in a stroke/TIA cohort. Our findings lay the foundation for a comprehensive understanding of post-stroke osteopathy and its clinical implications.

**Purpose:**

Patients who have suffered a stroke or transient ischemic attack are at high risk of fractures, which increase mortality and reduce health-related quality of life. Previous studies showed that ischemic stroke leads to unfavorable structural changes in the bones, but the underlying pathophysiological process is still unclear. Our aim was to analyze biomarkers and bone structure with high-resolution peripheral quantitative computed tomography (HR-pQCT) in a stroke/TIA cohort.

**Methods:**

This cross-sectional study analyzes the baseline data of a prospective cohort. Patients underwent HR-pQCT scans of the radius and tibia, alongside biochemical bone marker evaluation, within the first days after the acute cerebrovascular event.

**Results:**

Women had lower total, cortical, and trabecular volumetric bone mineral density (vBMD) and lower values for most other HR-pQCT parameters. Patients with increasing age had lower total and cortical vBMD, but no difference in trabecular parameters. Active or former smokers had lower total and trabecular vBMD. Higher BMI was associated with greater cortical and trabecular bone areas and higher trabecular density. A positive correlation was found between sclerostin levels and trabecular parameters. Higher osteoprotegerin levels were associated with lower cortical area and thickness. Osteocalcin showed a negative correlation with total vBMD and cortical thickness.

**Conclusions:**

This study is the first to combine HR-pQCT imaging with bone biomarkers to provide a detailed characterization of the structural and biochemical properties of bone in a stroke/TIA cohort. Our findings lay the foundation for a comprehensive understanding of post-stroke osteopathy and its clinical implications.

**Supplementary Information:**

The online version contains supplementary material available at https://doi.org/10.1007/s11657-026-01767-w.

## Introduction

There are over 100 million survivors of ischemic stroke worldwide, with an upward trend due to continuous aging of societies and declining stroke mortality [1]. Patients with ischemic stroke or transient ischemic attack (TIA) are at high risk of fractures. Prior research reports fracture rates of 22 to 74 per 1000 person years and up to sevenfold increased risk of hip fracture compared to the general population [2–10]. Fracture risk is highest in the first year after the ischemic event and gradually decreases over time [11]. Recent publications also show an elevated risk of fracture in the year before the ischemic event (with a further increase after the event) [3, 11, 12]. Post-stroke fractures increase mortality and reduce health-related quality of life [3, 11]. Neurological deficits, a high risk of falling, concomitant medications, and common risk factors of stroke and factures are possible causes for the increased risk of fracture, as is an alteration in bone structure after an ischemic event, termed “post-stroke osteopathy” [13, 14]. Previous findings using dual-energy X-ray absorptiometry (DXA) and, more recently, peripheral quantitative computed tomography (pQCT) and high-resolution peripheral quantitative computed tomography (HR-pQCT) show structural changes in bones after an ischemic stroke [14–21]. Although immobilization plays an important role, the underlying pathophysiological process of “post-stroke osteopathy” remains unclear. Former studies lack in sample size and prospective design and did not correlate structural changes of HR-pQCT parameters with biomarkers.

Bone strength is determined not only by bone mass, but also by bone morphology and microstructure [22]. This is where the limitations of planar DXA measurements lie. HR-pQCT is a tool for assessing cortical and trabecular bone mineral density and microarchitecture at peripheral skeletal sites with low radiation exposure [22].

## Purpose

The aim of this baseline analysis of the post-stroke osteopathy study is to characterize the cross-sectional bone microarchitecture and biochemical profile of our study cohort of patients with acute ischemic stroke or TIA. To our knowledge, this is the largest cohort of patients with ischemic stroke or TIA prospectively examined with HR-pQCT and the first study to correlate bone biomarkers with HR-pQCT parameters in this setting.

## Methods

**Fig. 1:**
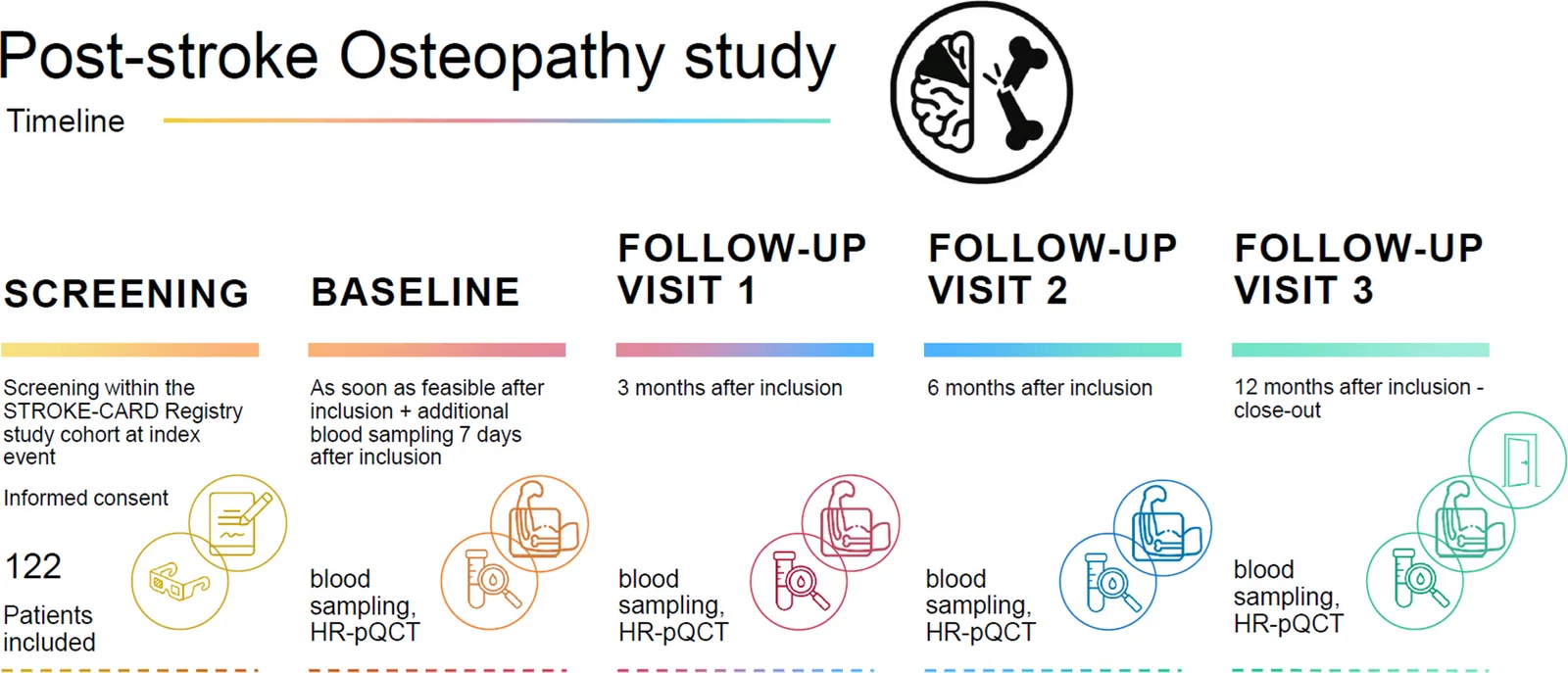
Timeline of the Post-stroke Osteopathy study; HR-pQCT, high-resolution peripheral quantitative computed tomography. The present paper reports analysis of the baseline data

The Post-stroke Osteopathy study (NCT04845269) is a pilot sub-study of the ongoing STROKE-CARD Registry (NCT04582825). Patients were enrolled from March 2021 to May 2022 at the University Hospital Innsbruck (Austria) (Fig. 1). All surviving patients with acute ischemic stroke and high-risk TIA (ABCD2 score ≥ 4) are invited to participate in the prospective STROKE-CARD Registry. It includes a baseline examination and follow-up examinations after 3 and 12 months, as previously described [23, 24]. Patients who participated in the STROKE-CARD Registry were eligible for inclusion in the Post-stroke Osteopathy study if they were between 60 and 85 years of age, had a modified Rankin Scale (mRS) < 5 at the time of inclusion, and gave written informed consent (specified in the study procedures of the post-stroke osteopathy study). Exclusion criteria were persistent motor deficit prior to the index event; inability to walk without a walking aid or to shift entire body weight to one of the legs prior the index event; history of stroke prior to the current ischemic event; premedication with corticosteroids for more than 6 weeks, or thiazolidinediones, bisphosphonates, or denosumab within the last 12 months; limb amputation; BMI < 18.5 kg/m^2^ or > 35 kg/m^2^; current or previous fracture affecting with HR-pQCT; and movement disorder affecting HR-pQCT imaging, and—as a standard regulatory requirement mandated by the local Ethics Committee—women of childbearing potential (though not applicable to this age group) were listed as excluded. All used scorings are further elaborated in the supplemental material.

For mentioned studies, participants provided their written informed consent. The studies were reviewed and approved by the ethics committee of the Medical University Innsbruck, Austria (STROKE-CARD Registry: 1182/2020, Post-stroke Osteopathy: 1504/2020).

In addition to the study procedures of the STROKE-CARD Registry, HR-pQCT imaging (XtremeCT II, Scanco Medical AG, Brütisellen, Switzerland; HR-pQCT scan parameters: 1467 mA, 68 kV, 900 projections/180° with an integration time of 43 ms per projection resulting in a 61 µm isovoxel size) of all four extremities (right and left distal radius, right and left distal tibia) was performed at baseline and 3, 6, and 12 months after the index event. The present paper reports the cross-sectional baseline data of the prospective, longitudinal cohort study. For this baseline analysis, only the initial HR-pQCT imaging was evaluated. Definition of the region of interest and motion grading were performed according to the guidelines [25]. This analysis comprises HR-pQCT imaging data recorded at baseline. Images with a motion grading of > 3, as recommended in the guidelines, were not included in the analysis (motion grading scale in the online supplement). For group comparisons, the mean value of right and left radius or the mean value of right and left tibia HR-pQCT measurements was used. To reduce data redundancy and enhance the clarity of presentation for the reader, we chose to pool the upper extremities and lower extremities.

**Fig. 2:**
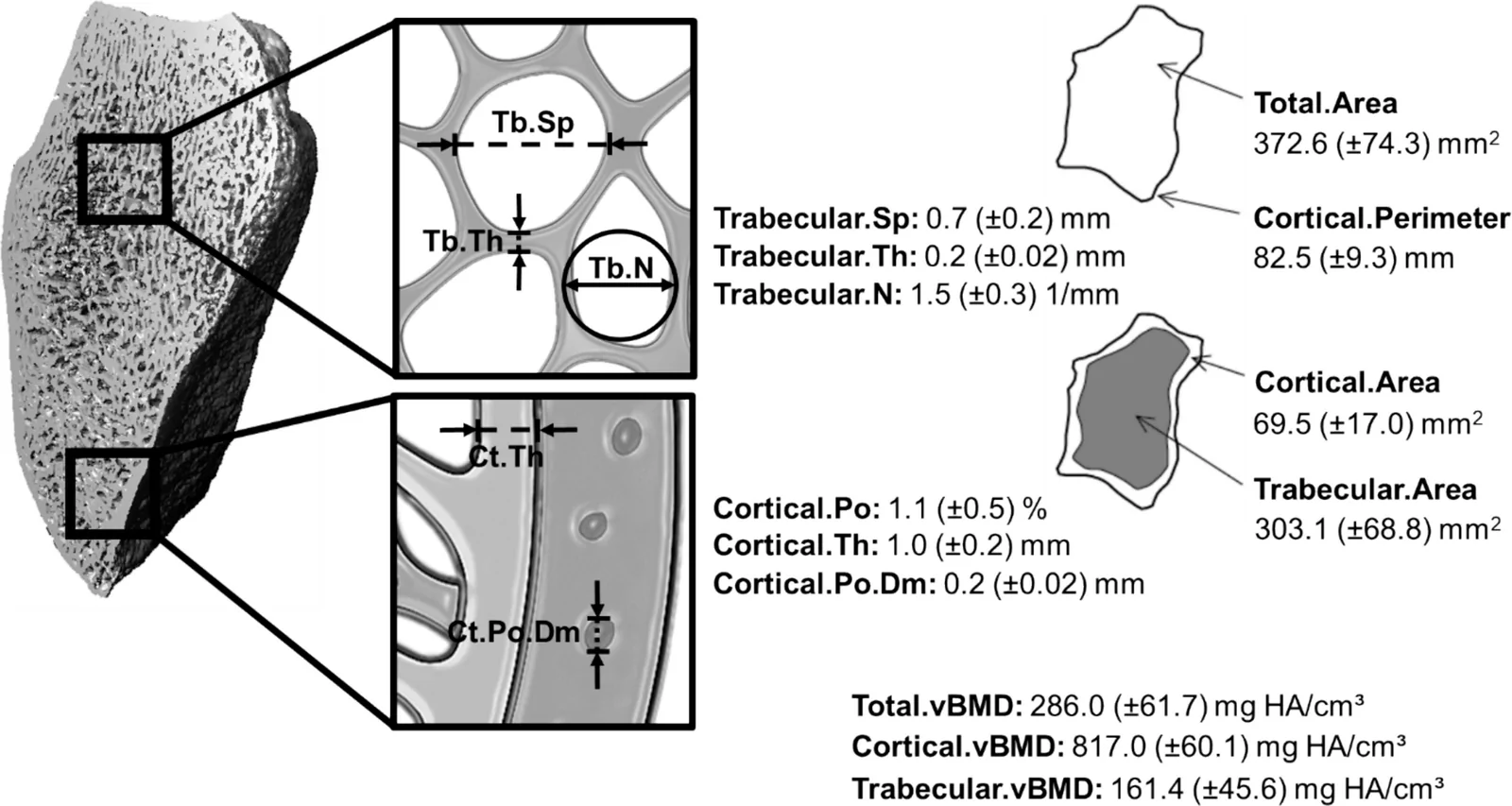
Schematic drawing of HR-pQCT imaging with mean (SD) values of our analyzed baseline data for the radius (see corresponding values and values for the tibia in Table 2); Tt.vBMD, total volumetric bone mineral density; Ct.vBMD, cortical volumetric bone mineral density; Ct.Ar, cortical area; Ct.Pm, cortical perimeter; Ct.Po, cortical porosity; Ct.Th, cortical thickness; Ct.Po.Dm, cortical pore diameter; Tb.vBMD, trabecular volumetric bone mineral density; Tb.Ar, trabecular area; Tb.N, trabecular number; Tb.Th, trabecular thickness; Tb.Sp, trabecular separation

Figure 2 shows a schematic drawing of HR-pQCT imaging. The analyzed parameters are defined as follows: total bone mineral density: average mineral density within the periosteal surface; cortical bone mineral density: average mineral density within the cortical compartment; trabecular bone mineral density: average mineral density within the trabecular compartment; total area: measure of total cross-sectional area within the periosteal surface; cortical perimeter: length of periosteal perimeter; cortical area: measure of the total cross-sectional area within the cortical bone compartment; trabecular area: cross-sectional area of the trabecular compartment; trabecular separation: average distance between trabeculae; trabecular thickness: average thickness of trabeculae; trabecular number: average number of trabeculae per unit length; cortical porosity: ratio of pore volume relative to the total volume of the cortical compartment, reported in percentage; cortical thickness: average thickness of the cortical compartment; cortical pore diameter: average 3D diameter of the pore volumes.

In addition to blood sampling for the STROKE-CARD Registry, blood samples for biomarker analysis were taken at five time points: as soon as feasible after the index event, 5 to 7 days after the index event, and at all follow-up visits (3, 6, and 12 months). For this baseline analysis, only the initial laboratory parameters were evaluated.

Serum samples (fasting) were analyzed by clinical standards via enzyme-linked immunosorbent assay (ELISA) for Osteocalcin (N-MID® Osteocalcin ELISA Immunodiagnostic Systems Limited, Boldon, United Kingdom; detection limit: 0.5 ng/mL; intra-assay coefficients of variation (CV) < 3.0%; inter-assay CV: < 5.0%), and C-telopeptide of crosslinked collagen type 1 [CTX-1] (Serum CrossLaps® (CTX-I) ELISA, Immunodiagnostic Systems Limited, Boldon, United Kingdom; detection limit: 0.020 ng/mL; intra-assay CV: < 3.0%; inter-assay CV: < 8.5%) at the Department of Internal Medicine 3—Rheumatology and Immunology, Uniklinikum Erlangen, Germany and for Sclerostin (Sclerostin ELISA, BI-20492; detection limit: 3.2 pmol/L; intra-assay CV: ≤ 7.0%; inter-assay CV: ≤ 5.0%), Osteoprotegerin [OPG] (OPG ELISA Kit (Human Osteoprotegerin) BI-20403 (detection limit: 0.07 pmol/L; intra-assay CV: ≤ 3.0%; inter-assay CV: ≤ 5.0%); soluble receptor activator of nuclear factor-κB ligand [sRANKL] (FREE soluble RANKL HS ELISA, BI-20462 (detection limit: 0.01 pmol/L; intra-assay CV: ≤ 5.0%; inter-assay CV: ≤ 3.0%) and Periostin (Human Periostin ELISA, BI-20433 (detection limit: 20 pmol/L; intra-assay CV: ≤ 3.0%; inter-assay CV: ≤ 6.0%) in the laboratory of Biomedica Bratislava, Slovakia all by Sandwich ELISA (HRP/TMB, 12 × 8-well detachable strips kits) produced by Biomedica Medizinprodukte Gmbh, Vienna, Austria [26–29]. For this analysis of biomarkers, samples drawn as soon as feasible after the index event (baseline sampling) were used.

Modified Rankin Scale (mRS) and National Institutes of Health Stroke Scale (NIHSS) were documented by certified physicians. Tissue-based definition of TIA was used. Body mass index (BMI) was calculated as weight in kilograms divided by the square of height in meters. Arterial hypertension, smoking status, and consumption of alcohol were recorded based on participants’ self-report or electronic patient records, as well as through in-hospital assessment. A standard drink was defined as 0.5 L of beer or 0.25 L wine or 2 cL distilled alcohol. If patients consumed no alcohol or less than two standard drinks per month, they were classified as non-alcohol consumers. Dyslipidemia was defined as low-density lipoprotein (LDL) > 100 mg/dL or high-density lipoprotein (HDL) < 40 mg/dL or total cholesterol > 200 mg/dL or lipoprotein(a) > 75 nmol/L or triglycerides > 150 mg/dL. Diabetes mellitus was defined as glycated hemoglobin (HbA1c) > 6.4 mmol/L in the first routine blood sample, as well as a known history of diabetes or already established medical treatment. Stroke/TIA etiology was categorized according to the TOAST criteria [30]. All variables mentioned were assessed in the baseline examination of the STROKE-CARD Registry, which was conducted during acute hospitalization after the index event.

### Statistical analyses

All statistical analyses were performed using SPSS version 27.0.1.0 (IBM SPSS Statistics, Armonk, NY, USA). Continuous variables are presented as mean (± standard deviation [SD]) or median (interquartile range [IQR] as appropriate) and categorical variables as absolute numbers and percentages (%). Comparisons between subgroups (sex, smoking status, diabetes mellitus, hypertension, dyslipidemia, alcohol consumption) were performed using Pearson’s chi-square for binary and nominal variables. Associations between continuous variables were assessed using Pearson’s correlation coefficient. To account for potential confounders, multivariable linear regression analyses were performed with adjustment for age and sex. A two-sided *p*-value < 0.05 was considered statistically significant. HR-pQCT parameters and biomarkers were dependent variables. In the correlation between HR-pQCT and biomarkers, the HR-pQCT parameters were dependent variables. The analyses were exploratory in nature. Due to the hypothesis-generating character of these secondary analyses, no formal adjustment for multiple testing was performed, and the results should be interpreted with appropriate caution. Beta coefficient represents the unstandardized regression coefficient. It indicates the change in the dependent variable (e.g., HR-pQCT bone parameter) for every one-unit increase in the independent variable (e.g., age, biomarker value), keeping all other variables constant. A positive beta indicates a direct positive relationship, while a negative beta indicates an inverse relationship.

## Results

**Fig. 3:**
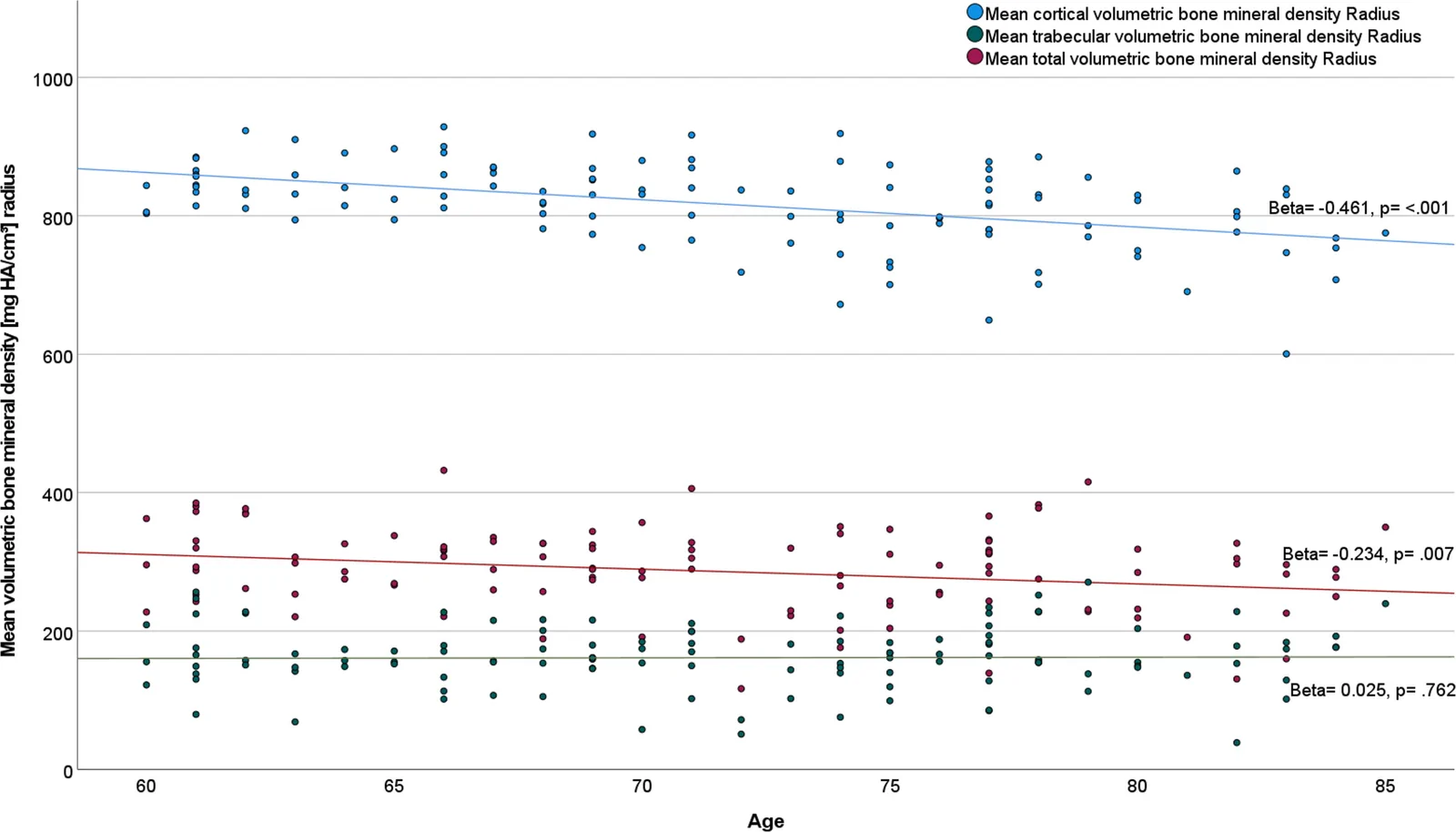
Correlation between age and volumetric bone mineral density in the radius; blue: cortical volumetric bone mineral density, red: total volumetric bone mineral density, green: trabecular volumetric bone mineral density; beta, beta coefficient; *p*, *p* value

One hundred twenty-two participants could be included in the study. In 116 patients, a HR-pQCT imaging was performed at baseline, with a median age of 71.5 (IQR 11.0) years, 26.7% were women, neurological deficits were predominantly mild, with a median NIHSS score of 2 (IQR 3), and 88.8% are right-handed (baseline characteristics are shown in Table 1). The mean time between admission and HR-pQCT was 5.4 (SD ± 2.3) days. There was no significant difference in total volumetric bone mineral density (vBMD) between radius of the dominant and non-dominant hand (284.9 [± 63.5] vs. 285.9 [± 66.1], *p* = 0.7). Women had a lower total vBMD, cortical vBMD (in tibia only) and trabecular vBMD, as well as lower values in almost all other HR-pQCT parameters (cortical area, cortical perimeter, cortical thickness, trabecular area, trabecular number, trabecular thickness, trabecular separation) (Table 2, Supplemental Fig. 1) in the upper and lower extremities. Patients with advanced age had a lower total and cortical vBMD, but no correlation with age in trabecular parameters could be found (Table 2, Fig. 3, Supplemental Fig. 1) in the radius and tibia. No correlation of any HR-pQCT parameter was found with diabetes mellitus, blood pressure, and dyslipidemia. A higher BMI correlated with a higher total vBMD, greater cortical area, and increased trabecular vBMD, trabecular number, and separation in the radius. In the tibia, a higher BMI was associated with a larger cortical perimeter and greater trabecular separation (Supplemental Table 1). Active or former smokers had no significant differences in the radius but a lower total and trabecular vBMD than non-smokers in the tibia (Supplemental Table 2). There was no correlation between NIHSS at admission or stroke etiology and HR-pQCT parameters. When excluding the 17 patients with TIA, findings did not change (data not shown).

**Table 1:** Baseline characteristics

	% or median (IQR) or mean (± SD)	Number
Age [years]	71.5 (11)	116
Women	26.7%	31
TIA	14.7%	17
Diabetes mellitus	17.2%	20
Hypertension	77.6%	90
Dyslipidemia	95.7%	111
Body mass index [kg/m^2^]	26.0 (± 3.3)	116
Handedness right	88.8%	103
Active smoking	21.6%	25
Active or former smoking	61.2%	71
Use of alcohol	63.8%	74
NIHSS at admission	2 (3)	116
Etiology by TOAST		
Large-artery atherosclerosis	19.0%	22
Cardioembolism	16.4%	19
Small-artery occlusion	24.1%	28
Other determined etiology	1.7%	2
Undetermined etiology	38.8%	45

*TIA *transient ischemic attack, *NIHSS* National Institutes of Health Stroke Scale, *TOAST* Trial of Org 10172 in Acute Stroke Treatment. There were no missing values

**Table 2:** Effects of age and sex on HR-pQCT parameters for radius and tibia

HR-pQCT parameter radius [unit]	All	Sex			Age	
		Women	Men	*p* value^a^	Beta coefficient	*p* value^b^
	*n* = 116	*n* = 31	*n* = 85			
Total bone mineral density [mg HA/cm^3^]	286.0 (61.74)	247.3 (58.52)	300.6 (56.70)	** < .001**	− 0.234	**.007**
Cortical bone mineral density [mg HA/cm^3^]	817.0 (60.09)	805.4 (75.44)	821.3 (53.05)	.198	− 0.461	** < .001**
Cortical area [mm^2^]	69.50 (16.98)	51.24 (10.06)	76.40 (13.62)	** < .001**	− 0.273	** < .001**
Cortical perimeter [mm]	82.47 (9.294)	72.23 (6.918)	86.34 (6.818)	** < .001**	0.099	.155
Cortical porosity [1]	0.011 (0.005)	0.010 (0.005)	0.011 (0.004)	.146	0.116	.220
Cortical thickness [mm]	1.006 (0.214)	0.858 (0.192)	1.062 (0.196)	** < .001**	− 0.347	** < .001**
Cortical pore diameter [mm]	0.185 (0.019)	0.182 (0.026)	0.186 (0.017)	.423	− 0.093	.330
Trabecular bone mineral density [mg HA/cm^3^]	161.4 (45.62)	123.7 (36.22)	175.7 (40.51)	** < .001**	0.025	.762
Trabecular area [mm^2^]	303.1 (68.83)	242.0 (54.85)	326.2 (58.89)	** < .001**	0.136	.087
Trabecular number [1/mm]	1.500 (0.284)	1.266 (0.272)	1.589 (0.234)	** < .001**	0.057	.487
Trabecular thickness [mm]	0.237 (0.019)	0.224 (0.014)	0.242 (0.018)	** < .001**	0.001	.992
Trabecular separation [mm]	0.674 (0.196)	0.831 (0.247)	0.614 (0.132)	** < .001**	− 0.018	.830
		Sex			Age	
HR-pQCT parameter tibia [unit]	All	Women	Men	*p* value^a^	Beta coefficient	*p* value^b^
	*n* = 116	*n* = 31	*n* = 85			
Total bone mineral density [mg HA/cm^3^]	277.6 (61.52)	233.9 (56.82)	294.2 (55.05)	** < .001**	− 0.160	**.060**
Cortical bone mineral density [mg HA/cm^3^]	793.1 (78.49)	749.2 (98.04)	809.7 (62.76)	** < .001**	− 0.332	** < .001**
Cortical area [mm^2^]	141.3 (36.76)	99.36 (20.39)	157.1 (28.06)	** < .001**	− 0.177	**.008**
Cortical perimeter [mm]	115.4 (11.64)	106.4 (9.673)	118.8 (10.49)	** < .001**	0.054	.517
Cortical porosity [1]	0.035 (0.012)	0.035 (0.013)	0.035 (0.012)	.900	0.064	.504
Cortical thickness [mm]	1.476 (0.364)	1.145 (0.255)	1.602 (0.317)	** < .001**	− 0.169	**.030**
Cortical pore diameter [mm]	0.224 (0.029)	0.217 (0.031)	0.226 (0.029)	.129	− 0.229	**.014**
Trabecular bone mineral density [mg HA/cm^3^]	172.8 (41.87)	149.3 (39.98)	181.7 (39.25)	** < .001**	− 0.015	.870
Trabecular area [mm^2^]	722.5 (164.4)	644.1 (146.4)	752.1 (161.9)	**.002**	0.049	.591
Trabecular number [1/mm]	1.398 (0.268)	1.289 (0.295)	1.439 (0.246)	**.007**	0.021	.818
Trabecular thickness [mm]	0.266 (0.022)	0.256 (0.019)	0.269 (0.021)	**.002**	0.032	.726
Trabecular separation [mm]	0.732 (0.209)	0.827 (0.320)	0.696 (0.132)	**.003**	0.000	.999

^a^adjusted for age, ^b^adjusted for sex; *HR-pQCT* High-resolution peripheral quantitative computed tomography, *HA,* hydroxyapatite, Beta coefficient: per standard-deviation unit

^a^adjusted for age, ^b^adjusted for sex, *HR-pQCT* High-resolution peripheral quantitative computed tomography, *HA*, hydroxyapatite

 In 62 patients, blood sampling at baseline was performed, with a median age of 73 (IQR 10.3) years and 27.4% were women (baseline characteristics shown in Supplemental Table 2). The mean time between admission and the first study-specific blood sample was 3.0 (SD ± 1.3) days. Women and younger patients had lower sclerostin levels (Supplemental Table 3). Higher OPG and Periostin but lower sRANKL levels were seen with increasing age (Supplemental Table 3). No differences in biomarkers were found between patients with and without diabetes mellitus, hypertension, dyslipidemia, and alcohol consumption (data not shown). BMI, NIHSS score, or stroke etiology showed no correlation with the biomarkers (data not shown).

A positive correlation was found between sclerostin levels and trabecular HR-pQCT parameters, including trabecular vBMD in radius and tibia. Patients with higher OPG levels had a lower cortical area in the radius. Osteocalcin showed a negative correlation with total vBMD and cortical and trabecular thickness in the radius, while in the tibia with cortical vBMD (Supplemental Table 4).

## Discussion

In this cohort of patients with cerebral ischemic events women had lower vBMD in the cortical and trabecular compartment, a finding consistent with former studies in the general population [31–33]. As previously shown, cortical and total vBMD is lower in older age, while the trabecular compartment appears to be less affected by age [33, 34]. The latter fact may be explained by higher Sclerostin levels in the elderly linked to stable trabecular parameters in our (Supplemental Table 3 and 4) and previous studies [35]. HR-pQCT parameters and sclerostin levels were examined for the first time in a stroke/TIA cohort.

Our data shows that smoking has a greater impact on the trabecular than on the cortical compartment, with lower total and trabecular vBMD in active and former smokers in the tibia. This is in line with previous findings [36]. Agarwal et al. showed an impact of smoking on the cortical compartment in women, while in men smoking had a greater impact on the trabecular compartment [37, 38].

We found a positive correlation between HR-pQCT parameters representing bone mass in the radius (total and trabecular vBMD, cortical area, trabecular number and separation) and BMI, which is consistent with former findings [39]. The effect of BMI was lower in the tibia. Although this lower impact on a weight-bearing bone is biomechanically counterintuitive, it underscores that BMI captures complex systemic processes rather than mere mechanical loading. It has been previously shown that dyslipidemia and high fat mass are associated with poorer bone quality and increased risk of fracture, and high cholesterol levels have a negative impact on the differentiation of bone marrow stromal cells [40–42]. In our cohort, these detrimental metabolic and lipotoxic pathways associated with higher BMI might have counteracted or masked the localized mechanical benefits typically expected at the tibia, leading to a less pronounced association at the lower extremity compared to the radius.

Previous studies have shown that higher osteocalcin levels correlate with increased bone turnover, particularly in conditions such as osteoporosis and trabecular thinning, reduced trabecular number, and increased bone fragility [43, 44]. Our data shows lower total vBMD, cortical, and trabecular thickness with increasing osteocalcin levels in the radius. Higher OPG levels were not associated with aberrations in trabecular HR-pQCT parameters, but correlated negatively with cortical area in the tibia. While this specific association has not yet been reported in a stroke/TIA cohort, it is in agreement with the STRAMBO study by Szulc et al. [36, 45], which similarly demonstrated a selective impact of elevated OPG on cortical rather than trabecular compartments [45]. Overall biomarkers had more impact on HR-pQCT parameters in the radius than in the tibia. As shown before, OPG levels increase with age [46].

## Limitations and strengths

This study has several limitations. The cohort was recruited at a single center. All participants were of European descent and enrolled in a European healthcare setting. In addition, the acute phase after ischemic stroke or TIA may influence biomarker levels, as may unmeasured confounding factors. Acute blood sampling at baseline was not feasible in all patients, which may have introduced additional variability and limits statistical power. HR-pQCT measurements were limited to peripheral skeletal sites and may not fully reflect changes in axial bone compartments. Furthermore, women were underrepresented due to eligibility criteria like exclusion of patients with known osteoporosis, which may have influenced sex-specific analyses. The correlations and analyses are exploratory, and given the large number of comparisons, the weak statistical significance observed in some parameters warrants a cautious interpretation. Furthermore, a limitation of the present cross-sectional analysis is the lack of an in-house, age-matched healthy control group. Consequently, our baseline findings remain exploratory and describe the absolute bone status of acute stroke/TIA patients.

Despite these limitations, to our knowledge, this study represents the largest cohort of acute stroke/TIA patients assessed prospectively with HR-pQCT to date and the first to combine HR-pQCT imaging data with bone biomarkers.

## Conclusion

Our findings provide a basis for future longitudinal studies aimed at clarifying the trajectory of bone changes after stroke or transient ischemic attack and elucidating the pathophysiology of “post-stroke osteopathy.”

## Supplementary Information

Below is the link to the electronic supplementary material.ESM 1(DOCX 94.2 KB)

## Data Availability

Data are available from Michael Knoflach upon reasonable request to the corresponding author, subject to a data sharing agreement.
