# Supplementary material for: High-resolution peripheral quantitative computed tomography imaging and bone biomarker characteristics of patients with ischemic stroke or transient ischemic attack—baseline analysis of the Post-stroke Osteopathy study
Source: Arch Osteoporos. 2026 Aug 29;21(1):134. doi: 10.1007/s11657-026-01767-w (PMC13525036; doi:10.1007/s11657-026-01767-w)
Supplement: Supplementary file 1 [file 11657_2026_1767_MOESM1_ESM.docx]

**Supplement**

**Methods**

STROKE-CARD Registry: After the STROKE-CARD trial demonstrated beneficial effects in terms of reducing cardiovascular events and improving health-related quality of life, the program was implemented as standard care in December 2020 by the Department of Neurology at the Medical University of Innsbruck and the St. John of God Hospital in Vienna, including an outpatient follow-up examination 3- and 12 months after the index event. Data from stroke and TIA patients were prospectively recorded in the STROKE-CARD Registry, which also includes patients with severe disability (mRS=5) at discharge. For the present analysis, data collected at the study center Innsbruck was used. The clinical information system of the University Hospital Innsbruck (KIS- PowerChart, Version: 2018.01, Cerner Corporation, 8779 Hillcrest Rd, Kansas City MO) allows hospital staff to document measures and record patient information (diagnoses, new and previous findings, new results, etc.). The Austrian electronic health records (ELGA database containing all available health related data in Austria from healthcare providers, i.e. hospitals, practitioners and pharmacies; introduced in hospitals in 2015 and for practitioners in 2019) and health records from the respective general practitioners.

HR-pQCT motion grading based on the guidelines of Whittier et al. (25):

1: No visible motion artifacts.
2: Very slight artifacts. Horizontal streaks at the upper and lower ends are visible.
3: Some artifacts. Horizontal streaks are visible, but cortex is intact.
4: Large horizontal streaks are visible. Cortex continuity is moderately disrupted at places and trabeculae are smeared.
5: Major horizontal streaks are visible. Complete disruption of continuity of the cortex and trabecular structure.

Clinical scoring methods:

To characterize the neurological status and etiology of the cohort, several standardized scores were used. The National Institutes of Health Stroke Scale (NIHSS) was recorded to assess the severity of neurological deficits based on 11 clinical parameters, including consciousness, vision, sensation, neglect, and motor strength (ranging from 0 to 42, with higher scores indicating greater impairment). For patients presenting with a TIA, the ABCD2 score was calculated to estimate early stroke risk based on five clinical parameters: Age (≥60 years), Blood pressure (≥140/90 mmHg), Clinical features (unilateral weakness, speech impairment), Duration of symptoms, and the presence of Diabetes (range 0-7). Functional independence was evaluated using the modified Rankin Scale (mRS), a 7-point scale ranging from 0 (no symptoms) to 6 (death), which measures the degree of disability in daily activities (higher scores indicate higher impairment). The ischemic stroke etiology was categorized according to the TOAST (Trial of Org 10172 in Acute Stroke Treatment) classification, which integrates clinical presentation, brain imaging (CT/MRI), and cardiovascular workups (ultrasound, ECG, echocardiography) to differentiate between large-artery atherosclerosis, cardioembolism, small-vessel occlusion, other determined etiology and undetermined etiology.

**Supplemental Tables**

Supplemental Table 1: Effects of BMI and smoking on HR-pQCT-parameters for radius and tibia

|  |  |  |  | BMI | |  | Smoking | | |
| --- | --- | --- | --- | --- | --- | --- | --- | --- | --- |
| HR-pQCT-parameter **Radius** [unit] |  | All n=116 |  | Beta coefficient | p value^1^ |  | Active or former smoking  n=71 | Never smoked  n=45 | p value^1^ |
| Total bone mineral density [mg HA/cm³] |  | 286.0 (61.74) |  | 0.181 | **.034** |  | 283.4 (62.11) | 289.8 (61.67) | .231 |
|  |  |  |  |  |  |  |  |  |  |
| Cortical bone mineral density [mg HA/cm³] |  | 817.0 (60.09) |  | 0.123 | .145 |  | 820.3 (52.22) | 811.8 (70.67) | .878 |
| Cortical area [mm²] |  | 69.50 (16.98) |  | 0.190 | **.004** |  | 69.68 (15.60) | 69.23 (19.07) | .363 |
| Cortical perimeter [mm] |  | 82.47 (9.294) |  | 0.086 | .220 |  | 82.64 (8.750) | 82.22 (10.16) | .852 |
| Cortical porosity [1] |  | 0.011 (0.005) |  | 0.009 | .923 |  | 0.011 (0.005) | 0.012 (0.005) | .312 |
| Cortical thickness [mm] |  | 1.006 (0.214) |  | 0.132 | .099 |  | 1.009 (0.203) | 1.001 (0.232) | .554 |
| Cortical pore diameter [mm] |  | 0.185 (0.020) |  | -0.045 | .637 |  | 0.183 (0.018) | 0.187 (0.023) | .282 |
|  |  |  |  |  |  |  |  |  |  |
| Trabecular bone mineral density [mg HA/cm³] |  | 161.4 (45.62) |  | 0.168 | **.042** |  | 157.8 (48.23) | 166.9 (41.28) | .110 |
| Trabecular area [mm²] |  | 303.1 (68.83) |  | 0.048 | .546 |  | 303.2 (64.22) | 302.9 (76.02) | .797 |
| Trabecular number [1/mm] |  | 1.500 (0.284) |  | 0.242 | **.003** |  | 1.479 (0.298) | 1.532 (0.260) | .139 |
| Trabecular thickness [mm] |  | 0.237 (0.019) |  | -0.010 | .908 |  | 0.236 (0.019) | 0.239 (0.019) | .246 |
| Trabecular separation [mm] |  | 0.674 (0.196) |  | -0.226 | **.006** |  | 0.689 (0.214) | 0.650 (0.165) | .119 |

|  |  |  |  | BMI | |  | Smoking | | |
| --- | --- | --- | --- | --- | --- | --- | --- | --- | --- |
| HR-pQCT-parameter **Tibia** [unit] |  | All n=116 |  | Beta coefficient | p value^1^ |  | Active or former smoking  n=71 | Never smoked  n=45 | p value^1^ |
| Total bone mineral density [mg HA/cm³] |  | 277.6 (61.52) |  | 0.091 | .285 |  | 268.6 (61.70) | 291.8 (59.18) | **.005** |
|  |  |  |  |  |  |  |  |  |  |
| Cortical bone mineral density [mg HA/cm³] |  | 793.1 (78.49) |  | 0.059 | .183 |  | 791.7 (77.11) | 795.4 (81.44) | .344 |
| Cortical area [mm²] |  | 141.3 (36.76) |  | 0.112 | .088 |  | 139.9 (36.05) | 143.4 (38.16) | .073 |
| Cortical perimeter [mm] |  | 115.4 (11.64) |  | 0.180 | **.032** |  | 116.5 (11.29) | 113.6 (12.07) | .270 |
| Cortical porosity [1] |  | 0.035 (0.012) |  | -0.118 | .219 |  | 0.035 (0.012) | 0.035 (0.013) | .931 |
| Cortical thickness [mm] |  | 1.476 (0.364) |  | 0.044 | .569 |  | 1.451 (0.364) | 1.517 (0.363) | .053 |
| Cortical pore diameter [mm] |  | 0.224 (0.029) |  | -0.082 | .374 |  | 0.223 (0.028) | 0.225 (0.032) | .409 |
|  |  |  |  |  |  |  |  |  |  |
| Trabecular bone mineral density [mg HA/cm³] |  | 172.8 (41.87) |  | 0.127 | .157 |  | 165.7 (41.87) | 183.9 (39.83) | **.006** |
| Trabecular area [mm²] |  | 722.5 (164.4) |  | 0.165 | .071 |  | 739.8 (161.6) | 695.3 (167.0) | .210 |
| Trabecular number [1/mm] |  | 1.398 (0.268) |  | 0.151 | .103 |  | 1.359 (0.274) | 1.500 (0.248) | **.026** |
| Trabecular thickness [mm] |  | 0.266 (0.022) |  | 0.003 | .976 |  | 0.264 (0.022) | 0.269 (0.021) | .149 |
| Trabecular separation [mm] |  | 0.732 (0.209) |  | -0.186 | **.043** |  | 0.763 (0.242) | 0.684 (0.129) | **.021** |

^1^adjusted for age and sex; HA: hydroxyapatite, BMI: body mass index; Beta coefficient: per standard-deviation unit; A preliminary three-group stratification separating current, former, and never smokers had been evaluated during initial data exploration but revealed no statistically significant differences in any HR-pQCT parameter.

Supplemental Table 2: Baseline characteristics of patient with blood sampling at baseline

|  | %  or  median (IQR)  or mean (±SD) | Number |
| --- | --- | --- |
| Age [years] | 73 (10.3) | 62 |
| Women | 27.4% | 17 |
| TIA | 19.4% | 12 |
| Diabetes mellitus | 12.9% | 8 |
| Hypertension | 77.4% | 48 |
| Dyslipidemia | 95.2% | 59 |
| Body mass index [kg/m²] | 25.8 (±3.4) | 62 |
| Active smoking | 25.8% | 16 |
| Active or former smoking | 72.6% | 45 |
| Use of alcohol | 67.7% | 42 |
| NIHSS at admission | 1 (3) | 62 |
|  |  |  |
| Etiology by TOAST |  |  |
| Large-artery atherosclerosis | 21.0% | 13 |
| Cardioembolism | 9.7% | 6 |
| Small-artery occlusion | 29.0% | 18 |
| Other determined etiology | 0.0% | 0 |
| Undetermined etiology | 40.3% | 25 |

TIA: transient ischemic attack, NIHSS: National Institutes of Health

Stroke Scale, TOAST: Trial of Org 10172 in Acute Stroke Treatment.

There were no missing values.

Supplemental Table 3: Effect of age and sex on bone biomarkers

|  |  |  |  | Sex | | |  | Age | |
| --- | --- | --- | --- | --- | --- | --- | --- | --- | --- |
| Biomarker [unit] |  | All n=62 |  | Women n=17 | Men n=45 | p value^1^ |  | Beta coefficient | p value^2^ |
| Sclerostin [pmol/l] |  | 37.53 (17.76) |  | 27.21 (8.977) | 41.43 (18.75) | **.009** |  | 0.316 | **.008** |
| OPG [pmol/l] |  | 5.744 (1.889) |  | 6.096 (1.886) | 5.614 (1.895) | .225 |  | 0.294 | **.021** |
| sRANKL [pmol/l] |  | 0.078 (0.050) |  | 0.079 (0.054) | 0.078 (0.050) | .911 |  | -0.260 | **.043** |
| Periostin [pmol/l] |  | 639.5 (221.5) |  | 578.7 (182.1) | 661.9 (232.2) | .286 |  | 0.275 | **.030** |
| Osteocalcin [ng/ml] |  | 14.48 (9.931) |  | 16.34 (10.00) | 13.78 (9.926) | .312 |  | 0.113 | .389 |
| CTX-1 [ng/ml] |  | 0.325 (0.158) |  | 0.341 (0.150) | 0.319 (0.162) | .659 |  | -0.021 | .874 |

^1^adjusted for age, ^2^adjusted for sex; OPG: Osteoprotegerin, CTX: C-telopeptide of crosslinked collagen; Beta coefficient: per standard-deviation unit;

Supplemental Table 4: Effects of bone biomarkers on HR-pQCT-parameters for radius and tibia

|  |  | Sclerostin [pmol/l] | |  | OPG [pmol/l] | |  | sRANKL [pmol/l] | |  | Periostin [pmol/l] | |  | Osteocalcin [ng/ml] | |  | CTX-1 [ng/ml] | |
| --- | --- | --- | --- | --- | --- | --- | --- | --- | --- | --- | --- | --- | --- | --- | --- | --- | --- | --- |
| HR-pQCT-parameter **Radius** [unit] |  | Beta coefficient | p value^1^ |  | Beta coefficient | p value^1^ |  | Beta coefficient | p value^1^ |  | Beta coefficient | p value^1^ |  | Beta coefficient | p value^1^ |  | Beta coefficient | p value^1^ |
| Total bone mineral density [mg HA/cm³] |  | 0.240 | .082 |  | -0.103 | .422 |  | 0.012 | .921 |  | 0.199 | .115 |  | -0.301 | **.015** |  | -0.237 | .054 |
|  |  |  |  |  |  |  |  |  |  |  |  |  |  |  |  |  |  |  |
| Cortical bone mineral density [mg HA/cm³] |  | 0.104 | .424 |  | -0.097 | .421 |  | -0.061 | .609 |  | 0.176 | .140 |  | -0.152 | .194 |  | -0.055 | .636 |
| Cortical area [mm²] |  | 0.202 | .065 |  | -0.141 | .169 |  | 0.065 | .519 |  | 0.137 | .177 |  | -0.119 | .234 |  | -0.195 | **.045** |
| Cortical perimeter [mm] |  | 0.118 | .240 |  | -0.102 | .276 |  | 0.141 | .123 |  | -0.056 | .550 |  | 0.219 | **.014** |  | 0.003 | .975 |
| Cortical porosity [%] |  | -0.039 | .795 |  | -0.148 | .289 |  | 0.169 | .218 |  | 0.081 | .561 |  | -0.044 | .746 |  | -0.046 | .732 |
| Cortical thickness [mm] |  | 0.143 | .280 |  | -0.101 | .403 |  | 0.006 | .959 |  | 0.245 | **.038** |  | -0.247 | **.036** |  | -0.226 | .052 |
| Cortical pore diameter [mm] |  | -0.068 | .654 |  | -0.177 | .204 |  | 0.288 | **.033** |  | 0.284 | **.038** |  | -0.040 | .770 |  | -0.009 | .948 |
|  |  |  |  |  |  |  |  |  |  |  |  |  |  |  |  |  |  |  |
| Trabecular bone mineral density [mg HA/cm³] |  | 0.292 | **.024** |  | -0.102 | .404 |  | 0.097 | .422 |  | 0.123 | .313 |  | -0.208 | .079 |  | -0.219 | .060 |
| Trabecular area [mm²] |  | 0.120 | .297 |  | -0.066 | .535 |  | 0.124 | .235 |  | -0.125 | .236 |  | 0.286 | **.005** |  | 0.041 | .689 |
| Trabecular number [1/mm] |  | 0.249 | .061 |  | 0.037 | .769 |  | 0.027 | .828 |  | 0.037 | .764 |  | -0.112 | .356 |  | -0.194 | .102 |
| Trabecular thickness [mm] |  | 0.288 | **.036** |  | -0.190 | .140 |  | 0.082 | .520 |  | 0.266 | **.037** |  | -0.330 | **.007** |  | -0.189 | .128 |
| Trabecular separation [mm] |  | -0.177 | .191 |  | 0.016 | .897 |  | -0.068 | .583 |  | -0.032 | .798 |  | 0.099 | .420 |  | 0.146 | .227 |

|  |  | Sclerostin [pmol/l] | |  | OPG [pmol/l] | |  | sRANKL [pmol/l] | |  | Periostin [pmol/l] | |  | Osteocalcin [ng/ml] | |  | CTX-1 [ng/ml] | |
| --- | --- | --- | --- | --- | --- | --- | --- | --- | --- | --- | --- | --- | --- | --- | --- | --- | --- | --- |
| HR-pQCT-parameter **Tibia** [unit] |  | Beta coefficient | p value^1^ |  | Beta coefficient | p value^1^ |  | Beta coefficient | p value^1^ |  | Beta coefficient | p value^1^ |  | Beta coefficient | p value^1^ |  | Beta coefficient | p value^1^ |
| Total bone mineral density [mg HA/cm³] |  | 0.228 | .092 |  | -0.127 | .293 |  | -0.009 | .940 |  | 0.061 | .619 |  | -0.153 | .202 |  | -0.172 | .143 |
|  |  |  |  |  |  |  |  |  |  |  |  |  |  |  |  |  |  |  |
| Cortical bone mineral density [mg HA/cm³] |  | 0.076 | .551 |  | -0.062 | .589 |  | 0.066 | .562 |  | 0.030 | .800 |  | -0.239 | **.030** |  | -0.034 | .761 |
| Cortical area [mm²] |  | 0.162 | .127 |  | -0.195 | **.040** |  | 0.111 | .241 |  | 0.016 | .788 |  | -0.118 | .208 |  | -0.135 | .143 |
| Cortical perimeter [mm] |  | 0.253 | .057 |  | -0.022 | .856 |  | 0.128 | .283 |  | 0.003 | .978 |  | 0.136 | .250 |  | -0.052 | .657 |
| Cortical porosity [%] |  | -0.286 | .064 |  | -0.095 | .501 |  | -0.167 | .227 |  | 0.002 | .990 |  | -0.108 | .434 |  | -0.001 | .994 |
| Cortical thickness [mm] |  | 0.052 | .679 |  | -0.213 | .053 |  | 0.028 | .799 |  | -0.043 | .699 |  | -0.204 | .060 |  | -0.130 | .229 |
| Cortical pore diameter [mm] |  | -0.189 | .197 |  | -0.143 | .277 |  | -0.108 | .406 |  | 0.099 | .453 |  | -0.176 | .172 |  | -0.012 | .927 |
|  |  |  |  |  |  |  |  |  |  |  |  |  |  |  |  |  |  |  |
| Trabecular bone mineral density [mg HA/cm³] |  | 0.359 | **.012** |  | -0.033 | .800 |  | -0.014 | .916 |  | 0.119 | .367 |  | 0.011 | .933 |  | -0.220 | .078 |
| Trabecular area [mm²] |  | 0.230 | .113 |  | 0.031 | .812 |  | 0.136 | .293 |  | 0.019 | .883 |  | 0.146 | .255 |  | -0.030 | .810 |
| Trabecular number [1/mm] |  | 0.376 | **.010** |  | 0.101 | .453 |  | 0.009 | .947 |  | 0.186 | .168 |  | 0.200 | .127 |  | -0.127 | .329 |
| Trabecular thickness [mm] |  | 0.314 | **.034** |  | -0.077 | .569 |  | -0.010 | .941 |  | 0.007 | .958 |  | -0.137 | .302 |  | -0.142 | .273 |
| Trabecular separation [mm] |  | -0.244 | .098 |  | -0.132 | .321 |  | -0.018 | .894 |  | -0.244 | .091 |  | -0.192 | .139 |  | 0.070 | .944 |

^1^adjusted for age and sex; OPG: Osteoprotegerin, CTX: C-telopeptide of crosslinked collagen; Beta coefficient: per standard-deviation unit;

**Supplemental Figures**

Supplemental Figure 1: Correlation between age and volumetric total bone mineral density separated by sex; pink/circle: women, blue/square: men; Beta: beta coefficient, p: p value.
